# Boosting inpatient exercise after hip fracture using an alternative workforce: a mixed methods implementation evaluation

**DOI:** 10.1186/s12877-024-04730-x

**Published:** 2024-02-14

**Authors:** Marie K. March, Sarah M. Dennis, Sarah Caruana, Christopher Mahony, James M. Elliott, Stephanie Polley, Bijoy Thomas, Charlie Lin, Alison R. Harmer

**Affiliations:** 1 Physiotherapy Department, Blacktown Mt Druitt Hospitals, Western Sydney Local Health District, Blacktown, NSW Australia; 2 Sydney School of Health Sciences, Faculty of Medicine and Health, University of Sydney, Camperdown, NSW Australia; 3 South Western Sydney Local Health District, Liverpool, NSW Australia; 4 Ingham Institute of Applied Medical Research, Liverpool, NSW Australia; 5 Physiotherapy Department, Hornsby Ku-ring-gai Hospital, Northern Sydney Local Health District, Hornsby, NSW Australia; 6 The Kolling Institute, St Leonards, NSW Australia; 7 Department of Rehabilitation and Aged Care, Blacktown Mt Druitt Hospitals, Western Sydney Local Health District, Blacktown, NSW Australia; 8 Department of Aged Care, Hornsby Ku-ring-gai Hospital, Northern Sydney Local Health District, Hornsby, NSW Australia; 9 Department of Orthopaedic Surgery, Blacktown Mt Druitt Hospital, Western Sydney Local Health District, Blacktown, NSW Australia; 10 Department of Orthopaedic Surgery, Hornsby Ku-ring-gai Hospital, Northern Sydney Local Health District, Hornsby, NSW Australia

**Keywords:** Hip fracture, Physiotherapy, Physical therapy, Health services research, Implementation science, Workforce, Mixed methods research

## Abstract

**Background:**

Hip fracture has a devastating impact on individuals and is an increasing burden for health systems and society. Compared to usual care, increased physiotherapy provision has demonstrated efficacy in improving patient and health service outcomes in this population. However, physiotherapy workforce challenges prevent sustained implementation.

**Methods:**

Our aim was to evaluate the safety, feasibility, acceptability, effectiveness and implementation cost of thrice daily physiotherapy for patients in the acute care setting after hip fracture at two public hospitals. We added twice-daily exercise implemented by an alternative workforce, to usual care consisting of daily mobility practice by a physiotherapist. Sites identified their preferred alternative workforce, with pre-registration physiotherapy students and allied health assistants chosen. We used a mixed methods approach, using the Consolidated Framework for Implementation Research (CFIR) as a determinant framework to guide implementation planning and data collection. We compared hospital length of stay data to a reference cohort.

**Results:**

We recruited 25 patients during the study period. Acute care hospital length of stay decreased from 11 days in the reference cohort to 8 days in the BOOST cohort (mean difference − 3.3 days, 95%CI -5.4 to -1.2 days, *p* = 0.003). Intervention fidelity was 72% indicating feasibility, no safety concerns were attributed to the intervention, and uptake was 96% of all eligible patients. The intervention was acceptable to patients, carers and healthcare providers. This intervention was cost-effective from the acute orthopaedic service perspective.

**Conclusion:**

Higher daily frequency of physiotherapy can be safely, feasibly and effectively implemented by an alternative workforce for patients in the acute care setting following hip fracture surgery.

**Supplementary Information:**

The online version contains supplementary material available at 10.1186/s12877-024-04730-x.

## Contributions to the literature

- There is demonstrated efficacy of high frequency exercise for patients in the acute stages after hip fracture, however no models of successful “real world” implementation have been described. This is the first published study to explore implementation of a high frequency exercise program for patients in the acute stages after hip fracture.
- Our approach, using the Consolidated Framework for Implementation Research with local stakeholder consultation, provided an innovative workforce solution to successfully implement high frequency physiotherapy for patients in the acute care setting after hip fracture, which can be translated to other clinical contexts.
- Our findings demonstrate the value of integrating Senior Physiotherapist and Clinical Educator roles in the acute orthopaedic setting to provide, safe, acceptable and effective, high frequency exercise for patients.

## Background

Hip fracture is a common, yet devastating, event for frail older people. At 12-months following hip fracture, less than 50% of people return to pre-fracture mobility, 20% require residential aged care, and 25% of people die [1]. From a health services perspective, this patient group accounts for a large proportion of hospital presentations and bed days, with over 44,000 Australian hospital separations accounting for 430,130 hospital bed days in 2019-20 [2]. Estimated costs to the health care system have approached a staggering $350 million AUD per annum in 2016 [3]. Incidence and related costs are projected to increase with our ageing populations worldwide [4]. This presents a challenge for health systems striving to maintain high-quality care despite an increasing census of patients.

A cardinal feature of high quality care for hip fracture includes mobility practice, usually conducted by physiotherapists [5, 6]. Initiating mobility practice within 24–36 h of surgery, and daily mobility practice over a seven-day week has been shown to reduce mortality and reduce hospital length of stay [7, 8]. However, a recent randomized controlled trial demonstrated higher daily frequency of physiotherapy in the acute care stage after hip fracture resulted in reduced overall hospital length of stay by nine days with no adverse effects [9]. As such, this is likely to be a high value, fiscally responsible, intervention towards reducing hospital bed days via a low cost post-surgical physiotherapy program [9, 10].

To our knowledge, despite evidence of efficacy, there exists a paucity of evidence towards exploring barriers or facilitators of implementing higher daily frequency physiotherapy in the acute care setting for patients after hip fracture. Volkmer and colleagues used qualitative methods to explore variation in service delivery by physiotherapists in the acute care stage after hip fracture [11]. They identified multiple barriers to best-practice physiotherapy, including the need for individualised care and a lack of physiotherapy staffing. In contrast, Snowdon and colleagues demonstrated that direct supervision of junior physiotherapists improved clinician adherence to early mobility guidelines after hip fracture [12], demonstrating that a facilitator of evidence-based care is the presence of senior physiotherapy staff at each individual service. There are also workforce-level barriers within physiotherapy around staff recruitment and retention [13]. Exploring alternative workforce models to support physiotherapy interventions is important for sustainability of high frequency therapy. Alternative workforce models include the use of allied health assistants or pre-registration physiotherapy students, to effectively substitute or safely enhance existing physiotherapy service provision at lower cost to health services [14–16]. Simultaneously this approach benefits students in terms of learning opportunities, and patients in terms of increased opportunity for therapy.

Given the demonstrated efficacy of higher daily frequency of physiotherapy after hip fracture [9], we used the Sax Institute’s Translational Research Framework [17] and framed our study to explore the replicability and adaptability of implementing higher daily frequency of therapeutic exercise in the acute care setting after hip fracture. The Consolidated Framework for Implementation Research was used to provide a systematic approach to implementation planning and qualitative assessment. The aim of this study was to determine if a higher daily frequency exercise program implemented by an alternative workforce for patients in the acute care setting after hip fracture was safe, feasible, acceptable, effective and cost-effective for the health system.

## Methods

### Design

Pre-post mixed methods implementation evaluation of higher daily frequency of therapeutic exercise after hip fracture conducted at two public hospitals in metropolitan Sydney, Australia.

#### Inclusion/exclusion criteria

This intervention was implemented at the service level, and all patients presenting to the service with hip fracture during the implementation stage were considered for inclusion. Participants who underwent surgical fixation for their hip fracture and were prescribed partial weight bearing (50% body weight through the affected lower limb in standing) or more after surgery were eligible for the study. We excluded patients who: (i) were living in residential aged care and were transferred back to their usual residence as soon as medically stable; (ii) did not have operative fixation for their hip fracture; (iii) were limited in their ability to bear weight through their upper limbs or lower limbs after surgery; and (iv) were unable to ambulate a distance more than from bed to chair before the index hip fracture.

#### Intervention

We modified Kimmel and colleagues [9] effective high frequency intervention after hip fracture, by changing the mode of exercise and workforce used to deliver the intervention (Table 1).

**Table 1:** Intervention details

**Intervention**	Kimmel and colleagues	**BOOST study**
Usual care	Once daily mobility practice delivered by a physiotherapist	Once daily mobility practice delivered by a physiotherapist
Higher frequency intervention	Once daily mobility practice delivered by a physiotherapist	Once daily mobility practice delivered by a physiotherapist
	PLUS	PLUS
	Twice daily mobility practice delivered by an allied health assistant	Twice daily sit-to-stand exercises delivered by an alternative workforce

#### Implementation planning

The Consolidated Framework for Implementation Research (CFIR) [18] was used as a determinant framework to guide implementation planning. Determinant frameworks provide an explanation or describe influences on implementation outcomes, and this provided a structured approach to identify barriers and strategies to address them during implementation planning stage.

During the pre-implementation phase (Stage 1), a research steering group and two research teams were formed. Each research team had a Senior Physiotherapist project manager who led implementation planning with input from the research steering group, along with Senior Orthopaedic Physiotherapists, Physiotherapy Department Managers and the lead author. During implementation planning non-core components of the intervention were decided by local inpatient physiotherapy teams, and the CFIR was used to guide discussion around barriers and facilitators of successful implementation. Strategies to support implementation were then discussed and agreed among local physiotherapy teams. This implementation plan was then shared with the local orthogeriatric multidisciplinary teams for their input and approval, including geriatricians, orthopaedic surgeons, senior nursing staff and allied health staff. Local project managers then prepared and documented an implementation plan. For a six-week period prior to implementing the BOOST study intervention, in order to collect baseline data and test data collection procedures, we collected data on muscle strength and mobility on day 7 post-operatively among patients who presented with hip fracture.

Key aspects of implementation planning included.

- Identification of alternative workforce. Both sites elected to use pre-registration physiotherapy students as the main available alternative workforce.
- Orientation and training needs of the alternative workforce. This included orientation to the hospital and physiotherapy service, and specifically to the BOOST study. Training needs included: manual handling, identification of the deteriorating patient, documentation, incident and escalation procedures, data collection, and clinical handover.
- Intervention content. Both sites elected to provide alternative exercise programs including chair-based, bed-based and upper limb strength exercises to allow for patient preference. Both sites elected to set an intervention goal of thrice-daily intervention during weekdays only. Weekend service provision was limited to usual care due to availability of physiotherapists and alternative workforce, as well as clinical demand across the participating hospitals. BOOST patients were identified for weekend staff to provide increased frequency of care if the opportunity arose.
- Intervention timing. One site chose to have fixed times for implementation of exercise sessions for the alternative workforce to facilitate caseload management across the physiotherapy team. Each site also planned the start date for Stage 2 in view of other workforce stressors that may affect implementation.
- Caseload management. This included expectations of implementation during planned and unexpected staff absences, COVID-19 workforce demands, and prioritisation of the BOOST intervention within existing service priorities.
- Communication. Clear expectations were outlined regarding communication between clinicians and project officers regarding identification of BOOST patients, delegation of BOOST patients, BOOST service provision and discharge of BOOST patients.
- Use of funding. One site opted to used funding for an additional clinical educator to support the alternative workforce. In contrast, the other site used funding to support a project manager to facilitate successful implementation, adherence to study protocols and data collection.

The implementation phase (Stage 2) was a ten-week program at each site in 2021. The exact timing of the 10-week period was determined by each clinical site. We planned weekly contact between local project managers and a member of the research steering committee to ensure adherence to protocol, identify new barriers and/or facilitators to successful implementation and identify strategies to maximise implementation. All eligible patients and their identified carers were invited to provide survey and interview data collection on the patient and/or carer perspective of BOOST implementation. Interviews were conducted by a study investigator who, where possible, was not involved in providing the intervention, at a time and place convenient to the participant, including face-to-face and telephone methods. The survey included both open-ended and closed questions to minimise participant burden, and to facilitate collection of high quality data from a diverse patient group, with anticipated low health literacy levels among participants from one of the hospital sites [19], with potentially high drop-out rates. Patient-related physical performance outcome data was also collected during this time. We have reported patient and carer survey data only in this manuscript, with extended qualitative analysis to be reported separately (Lau et al., 2024 under review BMC Geriatrics).

During the post-implementation phase (Stage 3), project managers collected remaining quantitative data regarding health service outcomes. Qualitative methods (semi-structured interviews or focus groups) were used to collect data from the alternative workforce, project managers, physiotherapists, orthogeriatric clinical staff, and health service managers regarding their staff experiences of BOOST, reflections on the implementation process and participation in an implementation science project. Here we report survey data from staff on intervention acceptability and staff burnout using the Maslach burnout inventory [20], with extended qualitative interview data to be reported separately. The Maslach burnout inventory was used to determine if individual clinician burnout impacted implementation, particularly in view of local COVID19 restrictions in the hospital and community context.

#### Ethics and consent

Ethics approval for the study was granted by Western Sydney Local Health District Human Research Ethics Committee (2020/ETH02718). We implemented service-wide changes to the physiotherapy service so we did not recruit patients individually for the high-frequency physiotherapy intervention. However, all eligible patients and carers were approached by a study investigator during their hospital stay and asked to provide written informed consent for qualitative data collection to explore their patient and carer experience of the high frequency intervention. A study investigator provided information describing the study overall, expectations of involvement, and the opportunity to ask questions. Written informed consent was obtained before qualitative data collection. Staff who were approached to be interviewed as part of the Stage 3 evaluation were asked to provide informed consent specifically regarding qualitative data collection. A study investigator provided the opportunity to ask questions and written informed consent was obtained before qualitative data were collected.

#### Data collection and outcome measures

Sociodemographic and surgical data were recorded. These data are already collected as part of the Australia New Zealand Hip Fracture Registry data collection procedures and include age, sex, pre-admission mobility, anaesthetic risk score, weight-bearing status, pre-injury cognitive impairment, survival at 30 days, and survival at 120 days. In addition, we used the modified Iowa Level of Assistance Scale (mILOA) to measure pre-morbid mobility. The mILOA is a six-item scale of functional mobility adapted for use in hip fracture, which has been shown to be valid, reliable and responsive in the acute care hospital setting [14, 21].

The primary outcome measure was acute care hospital length of stay. We also collected rehabilitation length of stay, and total hospital length of stay to understand any follow-on effects of implementation. Total hospital length of stay is the sum of acute care length of stay and rehabilitation length of stay. Secondary outcomes are detailed in Table 2. The 30-second chair stand test (30CST), which measures the number of sit to stands within 30 s, is a feasible, valid and reliable measure of functional lower limb strength and power in older people [22, 23].

**Table 2:** Summary of implementation outcome measures

Outcome	Data collected
Effectiveness	Length of stay: (calendar nights from electronic medical records)
	Primary outcome: acute care ward length of stay
	Rehabilitation ward length of stay
	Total hospital length of stay
	Performance tests:
	* 30-s chair-stand test at Day 7 post-operatively
	* Modified Iowa Level of Assistance at Day 7 post-operatively
Safety	Adverse events:
	* Incident reports from existing standardised reporting mechanisms (Incident Information Management System) in NSW Health
	* Records of clinical emergency response system calls from medical records
	* Complication data from medical records
	* Intensive care admission data from medical records
	Discharge destinations:
	* Discharge destination from the acute care ward, rehabilitation ward (if relevant) and supports needed on discharge services, sourced from medical records
Acceptability	* Qualitative interview, focus group and survey data from patients, carers, and healthcare staff involved in the implementation, and/or providing orthogeriatric care
	Open and closed question survey data will be reported here, with qualitative interview and focus group data reported separately
Feasibility and uptake	* Physiotherapy occasions of service from existing standardised reporting mechanisms in NSW Health to measure adherence to intervention
	(This was compared to predicted occasions of service as per individual site implementation plans.)
	* Uptake—percentage of eligible patients who were provided the higher daily frequency intervention
	* Explanatory audit data from medical records regarding reasons for non-adherence to intervention (e.g. reasons for declining participation in session, early cessation of treatment)
Implementation cost (health service perspective)	* Hospital costs using existing data generated by local hospital clinical coding department based on ICD-10 diagnostic coding
	* Staffing costs/time for implementing non-physiotherapy workforce including supervision, upskilling, training incorporating existing requirements

We used the work of Proctor and colleagues [24] to define our implementation outcomes which are specified in Table 2.

Implementation costs were considered from a health services perspective. The orthopaedic ward context was similar at both sites. Implementation costs were considered in terms of paid staff enhancements, staff time to implement the intervention, staff time on communication and caseload management, training the alternative workforce, and implementation planning and monitoring. Costs related to staff time spent on project-specific aspects such as data collection, ethics applications, and research steering committee meetings were not included.

#### Sample size

Previous efficacy studies report a 10-day improvement in median hospital length of stay with higher frequency intervention [9], equivalent to an effect size of 0.28 from a baseline of 35 days, and a six-day improvement in interquartile range from a baseline of 15 days [9, 25]. In light of our modified intervention and alternative workforce, we assumed an improvement in median total hospital length of stay of seven days, equivalent to an effect size of 0.2. Using an equation developed by O’Keeffe and colleagues [26], assuming 90% power and 5% significance level, we required 34 patients in total. Accounting for 15% dropout, this required 40 eligible patients. We anticipated 12 eligible patients per month per site, and thus we planned to implement higher daily frequency exercise sessions for two months to recruit 40 patients.

#### Statistical analyses

Data were de-identified and pooled before analysis in SPSS. Count data, percentages and descriptive statistics were used to describe central tendency and variation for quantitative variables including demographics, safety, feasibility, and uptake. Acceptability was analysed using content analysis and percentages. The benchmarks of 60% uptake and 70% fidelity have been previously reported in implementation studies in a similar clinical context and these were used as a reference during analysis.

All hospital length of stay data is likely to have a positively skewed distribution, so we planned non-parametric Mann-Whitney-U testing to analyse all length of stay data [27]. However, once data analysis began, we found unequal variance and sufficient differences in shape of the distribution between our samples such that the planned Mann-Whitney U test would not be sufficiently robust, so we used Welch’s test instead, a t-test allowing for unequal variance [28].

Our reference cohort consisted of standardized patient-level data collected at both sites as part of the Australia New Zealand Hip Fracture Registry over two years. We filtered this data according to our inclusion criteria using standardised variables collected by the registry as follows:

1. pre-morbid residence (people in residential aged care excluded)
2. considering pre-morbid mobility (people who were bed bound or chair bound excluded),
3. post-operative weight bearing status (people who were non-weight bearing or touch weight bearing after hip fracture surgery were excluded).

We predicted that lower limb strength and functional mobility scores would be normally distributed so we planned to use independent t-tests to compare pre-implementation to implementation stages.

We used a mixed methods analysis with a qual ➔ QUANT approach. Survey data and themes of qualitative work will explain our quantitative findings, and complete qualitative data will be reported separately.

## Results

### Baseline data

We included data from 25 patient participants across two hospital sites who presented over a ten-week period. Funding and time constraints limited our recruitment time, and COVID19 lockdowns limited hip fracture presentations during the recruitment period.

Uptake data are presented in Table 3, with baseline characteristics for the BOOST and reference cohorts presented in Table 4. The majority of patients who were not eligible for inclusion was due to pre-injury residence in aged care, and post-operative weight bearing status. One patient who was eligible was excluded for safety reasons by the senior physiotherapist in light of the patient demonstrating aggressive behaviours. The BOOST and reference cohorts were largely similar on all baseline characteristics, with the BOOST cohort having a slightly higher proportion of male patients, and patients with an ASA score of 3.

**Table 3:** Uptake

	**Site 1**	**Site 2**	**Reference cohort**
Number of people with hip fracture presenting to ED during implementation phase	31	22	Not applicable
Number of people with hip fracture eligible for inclusion in BOOST	17	9	231
Included participants (n)	16	9	230

**Table 4:** Baseline characteristics

Variable	BOOST cohort	Reference cohort
Age (mean in years, SD)	81 (8.8)	80 (10)
Pre-injury mILOA score (mean, SD)	3.44 (51)	NA
Female	56%	70%
Pre-injury cognitive impairment	20%	17%
Right hip fracture	56%	52%
ASA^a^ 2	20%	20%
ASA^a^ 3	64%	55%

^a^ASA denotes the American Society of Anaesthesiologists score

### Primary outcome: acute care length of stay

The BOOST cohort had a statistically and clinically significant reduction in acute care hospital length of stay of 3 days compared to the reference cohort, and an improved standard deviation of 7 days (mean 8.2 days SD 4.0 vs. mean 11.5 days SD 10.8; mean difference − 3.3 days, 95%CI -5.4 to -1.2, t=-3.1, *p* = 0.003).

### Secondary outcomes

#### Effectiveness

On inspection of the data for length of stay, we excluded one outlier from the reference cohort who had a total hospital length of stay of greater than 400 days.

When analysing data for patients who attended inpatient rehabilitation, there was no significant difference in rehabilitation length of stay detected between our BOOST cohort (*n* = 22) and the reference cohort (*n* = 116) (mean 32.6 SD 23.1 vs. mean 28.5 SD 18.1; mean difference 4.2, 95%CI -6.5 to 14.9, t = 0.84, *p* = 0.43).

When examining the data for total hospital length of stay, we noted two participants in the BOOST cohort and four participants in the reference cohort who were considered outliers, as their total hospital length of stay was greater than 90 days. These outliers had a significant effect on the BOOST cohort mean and SD. The mean total length of stay was 37.5 days with these outliers, and 31.5 days without these outliers. We saw a nine day change in standard deviation with these outliers removed. Given our objective is to explore our intervention in the real world setting, we retained these outliers in our analysis. There was a clinically and statistically significant difference in total hospital length of stay between the BOOST cohort and reference cohort (mean 37.7 days SD 25.6 vs. mean 25.9 days SD 23.0, t = 2.211; mean difference 11.79 days, 95%CI 0.874 to 22.713, *p* = 0.035).

Significant differences were noted in discharge destination from the acute care setting between the BOOST participants and reference cohort, with 88% of the BOOST cohort being discharged to inpatient rehabilitation, compared to 60% of the reference cohort (Chi-square 9.884, df = 4, *p* = 0.042). 4% of both BOOST and reference cohorts were transferred to other acute care wards, and 8% were transferred to residential aged care. No BOOST patients were discharged directly home, nor died during the study period, however, from the reference cohort, 26% of participants were discharged directly home from the acute care setting, and 3% died.

We were unable to collect our planned six week baseline data on lower limb strength and mobility during our pre-implementation phase due to time and funding constraints. Using patient and carer information regarding pre-injury mobility, we calculated pre-morbid mILOA data. Our mean pre-morbid mobility score on the mILOA was 3.4 (SD 5.1), and on Day 7 post-operatively mean mobility score was 19.0 (SD 5.5). Data for 17 participants was available for the 30SCST, with mean score 4.6 (SD 2.2).

#### Safety

One incident report was noted regarding a skin tear. One patient spent one day in intensive care for monitoring. Twelve clinical emergency response calls were noted: 4 for aggression from one patient with acute mental health concerns, 3 for glycaemic control, 2 for hypertension/tachycardia, 1 febrile, 1 low urine output. Thirteen patients experienced complications, including cognitive impairment (*n* = 4), hypotension (*n* = 2), anaemia (*n* = 2), pneumonia (*n* = 2), hyperglycaemia (*n* = 1), low urine output (*n* = 1), and acute mental health symptoms (*n* = 1).

#### Uptake and feasibility

All eligible participants were identified by the research team with 96% uptake. One patient was excluded for safety concerns by the treating senior physiotherapist due to aggressive behaviour. One patient was withdrawn from the study at day 7 post-operatively due to an acute mental health episode and non-engagement with the intervention for seven days.

In total, 303 occasions of service were provided to BOOST patients over the planned ten-week recruitment period, which was 72% of predicted occasions of service based on implementation planning. Timing and funding constraints limited our recruitment period to the planned ten weeks, and this period coincided with local increases in COVID19 cases and subsequent community lockdowns, hence our recruitment target was not reached. Reasons for non-adherence to the intervention were coded, with proportions as follows (note multiple reasons on some occasions): patient preference 28%, physiotherapy service availability 31%, patient unwell 14%, system issue 11%, patient unavailable 20%.

#### Implementation costs

Project managers spent 16–26 h over the life of the project supporting implementation. Fourteen hours was spent on research-specific activity. Time spent by other physiotherapy staff supporting implementation had high variability depending on the size of the broader inpatient team involved, with a minimum of nine hours across all stages of the project.

We estimated savings from a health system perspective based on our acute care hospital length of stay data with the following assumptions:

- Cost of a single bed day in the acute care setting was AU$1360 [29].
- Cost of a single bed day in the rehabilitation setting was AU$995 [29].
- 130 hip fracture patients presenting at each hospital site per annum [1].
- 50% of these patients would be eligible for BOOST as per our inclusion criteria.
- 3-day mean improvement in acute care hospital length of stay for these patients.

Our estimated cost-savings per site, per annum were:

Sixty-five patients per year x 3 acute care bed days per patient = 195 bed days per year.

One hundred ninety-five bed days x $1360 per day = $265,200 AUD cost savings per year for each hospital site.

Cost of Physiotherapy Clinical Educator per annum to the hospital is AU $133,474 including 20% on-costs.

Estimated cost-saving:

**Table:** 

Savings from intervention	-	Cost of intervention	Cost–benefit
AU $265 200	-	AU $133 474	AU $131 726

#### Acceptability- patient data

Patient and carer experience survey data were obtained from 16 participants. We asked five questions, with a five-item Likert scale for answers. A short, descriptive phrase and a colour-coded faces scale was used to correspond to each answer. There was insufficient data to differentiate patient and carer perspectives so this data is presented together.

Patients and carers perceived that they were treated with kindness and respect by the alternative workforce (100% satisfied/very satisfied), they enjoyed the exercises (94% satisfied/very satisfied), and they were satisfied with the amount of therapy provided (78% satisfied/very satisfied). When patients were asked if they would recommend BOOST to family and friends, 87% were likely or very likely to recommend BOOST. Patient perception of pain was varied, with 69% satisfied/very satisfied regarding pain levels, but 13% were neutral, and 13% were unsatisfied.

The opportunity for free text responses was provided at the end of the survey. Sixteen comments were provided by 10 patients. Patients perceived positive effects of exercise, and of achieving their goals regarding improved mobility and discharge home. Patients also valued the communication skills demonstrated by the alternative workforce, as “students were good at listening”. Patients perceived a choice of different exercises as a facilitator of implementation, with barriers to implementation including pain, and not feeling “in the mood”.

#### Acceptability- staff data

We used survey data from 36 staff regarding burnout to account for individual barriers to implementation. We used one item from each domain of the Maslach burnout inventory: emotional exhaustion, depersonalisation, personal accomplishment, with validated cut-offs for burnout at “once per week.” No participants experienced depersonalisation, with 22% experiencing emotional exhaustion (*n* = 8, staff and alternative workforce in equal proportion), and 22% experiencing a lack of personal accomplishment (*n* = 8, 88% of these were staff). We concluded that individual burnout was not a significant barrier to implementation of BOOST.

Survey data from 36 staff demonstrated that most staff perceived increased exercise for hip fracture patients as a beneficial treatment (94% agree/strongly agree), and that patients benefited from the BOOST project (86% agree/strongly agree). Staff perceived no difference to discharge planning (75%). When considering if the BOOST project facilitated high quality routine care, most staff were in agreement or neutral (53% agree/strongly agree, 42% neutral). Similar agreement was noted for teamwork within the wider hip fracture care team during BOOST (53% agree/strongly agree, neutral 44%).

In free text survey responses from staff, themes emerged regarding inclusion criteria, patient-level barriers to implementation including pain and medical frailty, effects of implementation within the multidisciplinary team and among patients, and service-level barriers to implementation.

Qualitative data from staff, patients and carers was collected regarding implementation experiences using the CFIR as a guide. Themes from this data were consistent with survey data and will be reported elsewhere (Lau et al., 2024 under review BMC Geriatrics). In summary, this data reflected that the CFIR domains of intervention characteristics, characteristics of individuals, and process were the most relevant for successful implementation in our study.

#### Mixed methods triangulation

Our survey data from staff, patients and carers informed us that the content was acceptable, and that the intervention was perceived to improve patient’s mobility, and progress them toward their goal of discharge home. No individual factors were identified as barriers to intervention, and overall our implementation process was endorsed by the physiotherapy teams and multidisciplinary teams. This explained our high fidelity (72%) and our effective intervention (3-day reduction of acute care hospital length of stay).

## Discussion

Our study demonstrated that implementing a higher daily frequency of physiotherapy using an alternative workforce for patients in the acute setting after hip fracture surgery, was (i) effective, (ii) safe, (iii) feasible, (iv) acceptable, and (v) cost-effective at a small scale. There were no patient- or staff-reported safety issues related to the intervention; it was delivered with high fidelity, it was acceptable from all stakeholders, effective in reducing acute care hospital length of stay by 3 days, and cost-effective from a health services perspective.

Considering age and sex, our cohort was similar to previous populations. However, our incidence of pre-operative cognitive impairment (20%) was lower compared to the hip fracture population in Australia (36%, ANZHFR 2021). our reference cohort and other studies. This may be due to our exclusion of people residing in aged care before their index hip fracture, as they are more likely to have varying levels of cognitive impairments. In comparison to published data on similar cohorts, our participants had lower calculated pre-morbid mobility scores but similar day 7 post-operative mobility scores [9, 21, 30, 31]. This supports our assertion that our physiotherapy intervention, boosted by an alternative workforce, was effective in improving mobility in the acute care setting after hip fracture for those with significant pre-morbid limitations. Results from the 30-second chair-stand test indicated that lower limb strength was well below healthy age-based normative values [22, 23], despite our participants using their upper limbs for support during the test. Our results from day 7 post-operative scores indicate that this patient group were still at a high risk of falls [31], which supports the clinical recommendation for and delivery of inpatient rehabilitation.

Previous work did not show that physiotherapy intervention three times daily in the acute post-operative period improved acute care hospital length of stay beyond six days [9]. Our improvement in acute care hospital length of stay was in the context of a reference cohort of 11 days, and the BOOST cohort improved to eight days. This supports the idea that the majority of patients with hip fracture present with frailty and multimorbidity and require a 6–7 day acute care inpatient stay in order to considered medically stable and optimised for inpatient rehabilitation or supported discharge. Aiming for improvements in acute care hospital length of stay beyond this point may not be feasible due the slow recovery trajectory patients with hip fracture. Our study also demonstrated an improved standard deviation of acute care hospital length of stay (4.0 days to 11.8 days), which is important for health services concerned with minimising unwarranted clinical variation. It is likely that improved unwarranted clinical variation of acute care length of stay leads to improvements in patient flow within a hospital setting, as improved bed availability in the orthopaedic ward leads to less time spent in the emergency department waiting for transfer. Future research needs to quantify the effect of high frequency acute care exercise interventions on patient flow from the emergency department to the orthopaedic ward, accounting for local seasonal variations in staffing and patient presentations.

An important consideration alongside our research is those who were not able to benefit. Although we had good uptake among those who were eligible for inclusion, a significant proportion of patients presented who were excluded, many due to residing in residential care before their index fracture. Given the increased mortality for these patients, further consideration needs to be given to care pathways that consider goals of care and minimise risks of hospitalisation in the frailest elderly. Our qualitative data demonstrated cognitive impairment, frailty and pain were barriers to acceptability and engagement with our intervention, which reflects the current literature [32]. Future research needs to consider development of an integrated intervention combining higher daily frequency of exercise and cognitive retraining, using expertise from physiotherapy and occupational therapy [33]. This approach could improve engagement with (i) patients with cognitive impairment and (ii) their carers to address current variation in observed outcomes, which could provide foundation for a more equitable, and effective, service.

There was no difference in rehabilitation length of stay between the BOOST cohort and the reference cohort, yet when total hospital length of stay was compared, there was a statistically and clinically significant difference, with the BOOST cohort staying 12 days longer. This is an unexpected result compared to previous work and may be reflective of the final discharge destination of included participants. In contrast to the reference cohort, no participants in the BOOST cohort were discharged directly home, nor died during the study period. This may also be due to the effect of long stay outlier patients, who adversely skewed our BOOST cohort data. Although the financial cost of longer rehabilitation stays may outweigh the financial costs of shorter acute care stays, the improved availability of acute care orthopaedic bed days is important given the increasing demand from both elective and trauma orthopaedic patients. This is particularly true in the sites where our study was conducted, as these are mixed orthopaedic wards, catering for both trauma and elective populations. Future research needs to explore whether this pattern is observed in studies with larger sample sizes or fewer outliers, and in different hospital contexts.

Both sites in our study chose to predominantly use pre-registration physiotherapy students as the alternative workforce who provided our additional structured exercise intervention. Student-led allied health services are an emerging area of research, with mutual benefit for patients and carers, health services, training providers and students alike [16]. We designed the intervention to ensure that fidelity was achievable by the alternative workforce, accounting for a wide range of skill and experience levels. During implementation planning we identified key training needs for our alternative workforce to ensure safety and fidelity. Two domains; manual handling and identification of the deteriorating patient, were already part of usual orientation procedures for pre-registration physiotherapy students at each site. The additional domain of orientation to the BOOST project specifically, including training in standardised objective measures, was added to the orientation program with minimal burden on staff or students. The costs of providing clinical educators to health services are far outweighed by the potential for savings in financial costs and bed days. Our analysis also demonstrates the efficiency of having a dedicated orthopaedic clinical educator to supervise students in a ward-based model, rather than a team approach to clinical education and supervision. Allied health assistants were used sparingly to implement interventions in our study, but other studies have shown they are safe and effective in implementing similar interventions [9, 14]. Future research needs to explore workforce resources subject to local availability, and what training needs different healthcare workers have to provide a safe, acceptable, and effective intervention.

Our study had some limitations, including recruiting fewer patients than anticipated (*n* = 25 compared to *n* = 40), as our recruitment phase coincided with a local increase in the number of COVID19 cases and subsequent community lockdown, which may explain reduced presentations to hospital for this population. At one site, approximately half the patients enrolled presented over one weekend, which was a challenge for clinicians to maintain fidelity of increased frequency of intervention for a higher number of patients than usual. We were also unable to obtain sufficient pre-implementation data for the 30-second chair-stand test as local sites chose to optimise adherence to the BOOST protocol during the implementation stage, and time and funding constraints limited our ability to prolong the project further.

Future research needs to consider how higher daily frequency of therapeutic physiotherapy can be implemented in an equitable way across different contexts with local demands. Consideration of how mobility practice can be safely implemented with an alternative workforce warrants further exploration. Although we have focused on hip fracture care in this study, frail older people admitted to hospital in a variety of clinical areas for a variety of diagnoses with multiple comorbidities may benefit from higher daily frequency of exercise during hospitalisation to prevent deconditioning; and exploring the mode, frequency and implementation of exercise and physical activity in this population may have wide-ranging benefits.

## Conclusions

It is safe, feasible, acceptable, effective and cost-effective for an alternative workforce to implement high frequency exercise with patients in the acute care stage after hip fracture surgery at a small scale. This approach to successful implementation can be easily replicated in other settings. It is likely this approach is financially sustainable in our setting.

### Supplementary Information

**Additional file 1.** BOOST- Appendix.

## Data Availability

The datasets generated and/or analysed during the current study are not publicly available as they are considered private health information in our jurisdiction but may be available from the corresponding author on reasonable request.
