# Supplementary material for: Boosting inpatient exercise after hip fracture using an alternative workforce: a mixed methods implementation evaluation
Source: BMC Geriatr. 2024 Feb 14;24:149. doi: 10.1186/s12877-024-04730-x (PMC10865645; doi:10.1186/s12877-024-04730-x)
Supplement: Supplementary file 1 — Additional file 1. BOOST- Appendix. [file 12877_2024_4730_MOESM1_ESM.docx]

# BOOST- Appendix

Implementation costs from Physiotherapy Departments

|  | Cost from existing resourcing | Cost from research funding |
| --- | --- | --- |
| SITE 1 | - Level 3 Orthopaedic Physiotherapist - Three existing Level 4 Physiotherapy Clinical Educators (non-orthopaedic) | - Project manager: Level 3 Physiotherapist x 4 hours per week for 52 weeks |
| SITE 2 | - Project manager- Level 3 Physiotherapist (inpatient) - Level 3 Orthopaedic Physiotherapist - Level 1/2 Physiotherapist on orthopaedic ward | - Upgrade a Level 2 Physiotherapist to 1 x Level 4 Physiotherapy Clinical Educator 38 hours x 10 weeks |
| Please refer to the following document: [NSW HEALTH SERVICE HEALTH PROFESSIONALS (STATE) AWARD 2021](https://www.health.nsw.gov.au/careers/conditions/Awards/health-professional.pdf) for information regarding Physiotherapist roles and grading. | | |

| Staff member | Justification of time spent | Time spent |
| --- | --- | --- |
| Project manager- implementation planning | - Pre and post implementation planning meetings - Regular reviews during implementation phase - Documentation of meeting minutes and preparation of implementation plan | 6 hours total |
| Project manager- daily project management | - Project management and communication with stakeholders (variance depending on one-to one liaison vs large acute team) - Liaison with management = 2-3 hours total | 15-40 mins per day  2-3 hours total |
| Project manager- training preparation and delivery | - Preparation of training materials for alternative workforce and multidisciplinary team - Delivery of training:   Physiotherapy team and multidisciplinary team  Alternative workforce | 2-3 hours total  2-3 hours total  1-2 hours per five weeks |
|  | TOTAL | 16-26 hours |
| Project manager- research specific time | - Qualitative data collection - ICH Good Clinical Practice training - Review of ethics and governance applications | 6 hours total  4 hours total  4 hours total |
|  | TOTAL | 14 hours |
| Physiotherapy Clinical Educator/s and inpatient staff | Pre and post implementation planning meetings  Caseload management including delegation: multiple educators vs sole clinical educator  Informal communication regarding research project with research team (multiple educators vs sole clinical educator) | 3 hours per person total  30-300 mins per staff member per week of implementation  6-60 mins per week of implementation |
| Physiotherapy staff- research specific time | Review of ethics application, governance application, dissemination activities, data collection | 1.5 hours total |
